# Age and Gender Differences in Long-Term Exercise Behavior for Older Adults with Heart Disease

**DOI:** 10.1093/geroni/igab046.3243

**Published:** 2021-12-17

**Authors:** Helen Graham, Yuki Asakura, Kathy Prue-Owens

**Affiliations:** 1 University of Colorado Colorado Springs, Colorado Springs, Colorado, United States; 2 Centura Health Penrose - St. Francis, Colorado Springs, Colorado, United States

## Abstract

Exercise decreases mortality and hospital admissions. Exercise adherence is challenging, and little is known about exercise adherence especially in older adults with heart disease. To gain an understanding of long-term exercise behaviors in older adults we conducted a cross-sectional study of individuals diagnosed between 2016-2020 with myocardial infarction (MI)/angina. Emails were sent in 2020 to recruit participants. Exercise adherence was measured using the Exercise Adherence Rating Scale (EARS), Godin’s Leisure-Time Activity Scale (GLTEQ) for exercise intensity, and self-report for impact of COVID-19. Descriptive statistics and t-tests were used to analyze data. Eight-hundred and seven individuals (x ® age 67.3) responded to the on-line survey. The majority were males (68.8%), married, (68.9%), and retired (59.3%). Co-morbidities included hypertension (32%), hyperlipidemia (21%), diabetes (12%), and depression (6.2%). Long-term exercise behaviors were independently observed in participants ≥65yr (n=526) and <65yr (n=281). Females ≥65yo demonstrated higher exercise adherence scores compared with males ≥65yo (1.66 ± 1.1 vs. 1.30 ± 21.7; t = -2.59, p=.010). Conversely, males scored higher in exercise intensity (34.4 ± 24.7 vs. 22.6 ± 21.7; t = 3.84, p=.000). Gender related exercise adherence and exercise intensity did not differ significantly in <65yo (p=.278 & p=.282, respectively). Exercise frequency decreased in both age groups after COVID-19 Pandemic started, however the decrease was significant only in older adults (p=.014) indicating they were at greater risk for exercise problems when faced with environmental barriers. Additional research is recommended as to the impact of environmental factors on exercise adherence in older adults and potential interventions.

